# Lymphopenia Induced by Different Neoadjuvant Chemo-Radiotherapy Schedules in Patients with Rectal Cancer: Bone Marrow as an Organ at Risk

**DOI:** 10.3390/curroncol31100429

**Published:** 2024-09-25

**Authors:** Christos Nanos, Ioannis M. Koukourakis, Admir Mulita, Raphaela Avgousti, Vassilios Kouloulias, Anna Zygogianni, Michael I. Koukourakis

**Affiliations:** 1 Department of Radiotherapy and Oncology, Medical School, Democritus University of Thrace, University Hospital of Alexandroupolis, 68100 Alexandroupolis, Greece; c_nanos@hotmail.com (C.N.); admirm@hotmail.gr (A.M.); 2 Radiation Oncology Unit, Aretaieion Hospital, School of Medicine, National and Kapodistrian University of Athens, 11528 Athens, Greece; ikoukourakis@med.uoa.gr (I.M.K.); azygogianni@med.uoa.gr (A.Z.); 3 Medical Physics Unit, Aretaieion Hospital, School of Medicine, National and Kapodistrian University of Athens, 11528 Athens, Greece; raphaela.avgousti@gmail.com; 4 Department of Clinical Radiation Oncology, Attikon Hospital, School of Medicine, National and Kapodistrian University of Athens, 12462 Athens, Greece; vkouloul@med.uoa.gr

**Keywords:** rectal cancer, radiotherapy, hypofractionation, bone marrow, lymphopenia

## Abstract

Radiotherapy (RT)-induced lymphopenia may hinder the anti-tumor immune response. Preoperative RT or chemo-RT (CRT) for locally advanced rectal cancer is a standard therapeutic approach, while immunotherapy has been approved for mismatch repair-deficient rectal tumors. We retrospectively analyzed 98 rectal adenocarcinoma patients undergoing neoadjuvant CRT with VMAT (groups A, B, C) or IMRT (group D) techniques, with four different RT schemes: group A (n = 24): 25 Gy/5 Gy/fraction plus a 0.2 Gy/fraction rectal tumor boost; group B (n = 22): 34 Gy/3.4 Gy/fraction, with a 1-week treatment break after the first five RT fractions; group C (n = 20): 46 Gy/2 Gy/fraction plus a 0.2 Gy/fraction rectal tumor boost; group D (n = 32): 45 Gy/1.8 Gy/fraction followed by 5.4 Gy/1.8 Gy/fraction to the rectal tumor. We examined the effect of the time-corrected normalized total dose (NTD-T) to the BM on lymphopenia. Groups A and B (hypofractionated RT) had significantly higher lymphocyte counts (LCs) after RT than groups C and D (*p* < 0.03). An inverse association between the LCs after RT and NTD-T was demonstrated (*p* = 0.01). An NTD-T threshold of 30 Gy delivered to 30% of the BM volume emerged as a potential constraint for RT planning, which could be successfully integrated in the RT plan. Hypofractionated and accelerated RT schemes, and BM-sparing techniques may reduce lymphocytic damage and prove critical for immuno-RT clinical trials.

## 1. Introduction

Rectal adenocarcinoma is a common human malignancy. The estimated number of new cases in the United States is 44.850 per year, with a male-to-female ratio of 1.5 [1]. The treatment of locally advanced disease (LARC) is under continuous evaluation and revision, with neoadjuvant chemo-radiotherapy (CRT) being a widely applied treatment modality [2]. Although the pathological complete response rates do not exceed 30%, the recent OPRA trial suggested that 74% of patients receiving total neoadjuvant therapy reach a complete or near complete clinical response. Moreover, when a “watch-and-wait” approach was applied, the 3-year total mesorectal excision-free survival rate was 53% in patients treated with concurrent CRT followed by consolidation chemotherapy [3].

New treatment approaches that enhance the efficacy of radiotherapy (RT) and chemotherapy could potentially render rectal cancer a medically curable disease, limiting the need for surgery. Immune checkpoint inhibitors (ICIs) seem to greatly contribute to this aim, as mismatch repair (MMR)-deficient tumors are already treatable with upfront anti-PD-1 monoclonal antibodies, limiting the necessity of CRT to patients with an incomplete response [4]. The combination of immunotherapy with CRT in the neoadjuvant setting for patients with LARC MMR-proficient patients is also under intense investigation [5]. Phase II protocols using long course RT, however, have shown a pCR rate between 22 and 33%, which is rather similar to the one obtained with CRT alone.

A robust immune system is certainly essential for the effectiveness of immunotherapy. Although tumor irradiation triggers antitumor immune responses [6], eventually enhancing ICI efficacy, clinical RT acts a double-edged sword. The lymphotoxic effect of RT and its impact on RT efficacy has been addressed in many published studies [7]. Severe lymphopenia has been associated with poor tumor response after neoadjuvant CRT in LARC [8]. Thus, it is postulated that RT-induced lymphopenia may hinder ICI efficacy, decreasing the expected benefit from immunotherapy–RT combinations. Although there are no clinical data to support this hypothesis in rectal cancer, a recent meta-analysis on 1130 lung cancer patients treated with ICIs showed that treatment-related lymphopenia was associated with a doubled risk of progression [9].

In this study, we examined RT-related parameters, namely dose fractionation, treatment acceleration, and bone marrow exposure to radiation, for their lymphotoxic relevance. Suggestions for the optimal RT schedule and demanded adjustments to the RT planning to decrease the risk of lymphopenia are provided.

## 2. Materials and Methods

We report a radiobiological analysis of 98 patients who underwent preoperative CRT for LARC treated at the Radiotherapy/Oncology Department of the University Hospital of Alexandroupolis (groups A, B, and C) and at the Radiation Oncology Unit of Aretaieion Hospital of Athens (group D). All patients had MRI-confirmed T3-stage and node-positive disease. Patients with a tumor extension to the anal region were excluded. This retrospective study has been approved by the Ethics and Research Committees of the two hospitals (approval numbers ES10 24-10-2018 and 316/26-03-2021, respectively). Written informed consent was obtained by all patients who gave permission to use their clinical and laboratory data anonymously for research purposes.

### 2.1. Radiotherapy Technique

The recruited patients were divided into four groups according to the applied RT fractionation. Group A (24/98 patients) was treated with the ultra-hypofractionated RT (ultra-HypoAR) scheme [10], and received 25 Gy (5 Gy/f) to the rectum and pelvic lymph nodes (5 daily fractions for 5 consecutive days). A simultaneously integrated boost (SIB) of 0.2 Gy was applied to the radiologically detectable rectal mass, as also reported by other study groups [11,12]. Group B comprised 22/98 patients treated with a hypofractionated accelerated RT (HypoAR) schedule delivering 34 Gy (3.4 Gy/f) to the rectum and pelvic lymph nodes in 10 fractions, 5 fractions per week, within 19 days (a break of one week was inserted after the first 5 fractions) [13]. Group C (20/98 patients) was treated with a simultaneous integrated boost (SIB) conventional RT scheme that applied 50.6 Gy (2.2 Gy/f) to the rectum and 46 Gy (2 Gy/f) to the pelvic lymph nodes in 23 fractions, 5 fractions per week, within 31 days. Group D (32/98 patients) was treated with a conventionally fractionated two-phase RT schedule delivering 45 Gy to the rectum and pelvic lymph nodes (1.8 Gy per fraction, for 25 fractions, 5 fractions per week, within 33 days). A booster RT dose was thereafter delivered in 3 fractions of 1.8 Gy to the rectal mass. The aforementioned RT schemes are utilized in the standard clinical practice of the Radiation Oncology departments at the mentioned University Hospitals.

Groups A, B, and C were treated with the image-guided volumetric modulated arc RT technique (VMAT/IGRT, 6MV ELEKTA InfinityTM Linear Accelerator (Stockholm, Sweden). The treatment plans were created using Monaco TPS version 5.11.03 (Elekta CMS, Maryland Heights, MO, USA). Each PTV was planned to receive at least 95% of the prescription dose to 98% of its volume. Before every treatment of each patient, a cone-beam CT (CBCT) was performed by XVI system (Elekta platform Synergy) for checking and adjusting patient position. Patients of group D were treated with an intensity-modulated IMRT technique. RT was delivered via a 6MV linear accelerator (Siemens Oncor Impression, Forchheim, Germany), and treatment planning was performed using Oncentra TPS version 4.5.3.1 (Elekta).

Patients in groups A and B received intravenous 5FU (600 mg/m^2^ bolus) and oxaliplatin (100 mg/m^2^) chemotherapy on the day of the first RT fraction and the 6th RT fraction (for group B). Patients in groups C and D received daily capecitabine chemotherapy (825 mg/m^2^ twice a day for 5 days per week) during RT, starting on the first day of RT. According to the protocol, patients of groups A, B, and C continued chemotherapy in the context of total neoadjuvant therapy, while patients in group D were referred for surgery.

### 2.2. Structure Delineation

All patients were scanned with a computed tomography (CT) simulator in a supine position with an immobilization device for the knees. Instructions for a comfortable full bladder and an empty rectum were given to each patient before simulation. The CT images were transferred to the Treatment Planning System (TPS) for structure delineation and treatment plan production.

The clinical target volume (CTV) includes the rectum, the internal and common iliac nodes up to the lower margin of the fifth lumbar vertebra, and the internal obturator and the presacral nodes. CTV margins for the creation of the planning target volume (PTV) were set at 1 cm laterally, anteriorly, and posteriorly, followed by manual correction where necessary. The CTV of the boost was defined as the radiologically detectable tumor–gross tumor volume (GTV) plus a margin of 2 cm, while a margin of 0.5 cm beyond CTV was considered for PTV with manual adjustment. The delineation of the area to receive the booster dose also took into account the MRI imaging, but we did not use any CT/MRI image fusion. No booster dose was prescribed to enlarged pelvic nodes. The bladder, sigmoid, and small intestine were contoured as organs at risk (OARs).

For this study, the bone marrow (BM) structure was contoured retrospectively, as this OAR had not been included in the original protocol. Adjusted skeletal windows of CT were applied to help the delineation of BM. This concerned the internal bone areas encompassed by the dense peripheral bone tissue. BM contouring was performed from the fifth lumbar vertebra to the lesser edge of trochanters, including all bone structures.

### 2.3. Radiobiological Analysis

For each patient, the raw data of cumulative physical dose–volume histogram (pDVH) of BM were extracted to an Excel spreadsheet. By applying the formulas presented below, at each point of the above DVHs [14], biological DVHs (bDVHs) were created. This procedure has been published in previous studies from our departments [15].

The Normalized Total Dose (biological dose) formulas without and with time correction (*NTD* and *NTD*_*T*), also known as Equivalent Dose in 2 Gy (EQD2), are

(1) $$NTD\left(a/\beta \right)=D\cdot \frac{\frac{\alpha }{\beta }+d}{\frac{a}{\beta }+2\;Gy}$$

(2) $$NTD\_T\left(a/\beta \right)=D\cdot \frac{\frac{\alpha }{\beta }+d}{\frac{a}{\beta }+2\;Gy}\;+\;\lambda \;(Tc\;-\;To)$$

where *D* is the total physical dose, *d* is the physical dose per fraction, *α*/*β* is the ratio that provides the dose in Gray where cell killing from linear and quadratic components of the linear–quadratic equation are equal, λ is the estimated daily dose consumed to compensate for rapid tumor repopulation, Tc is the number of days required for the delivery of the NTD using conventional fractionation, and To is the number of days required for the delivery of the accelerated scheme.

Considering that the BM is an early-responding tissue, an analysis for early toxicity was performed for an *α*/*β* ratio of 10 and 15 Gy. Also, a λ-value of 0.2 Gy/day was considered to adjust for the overall treatment time (OTT) acceleration [16]. Five dose points of the bDVHs were chosen for comparisons, namely, D80%, D50%, D30%, D20%, and D10%, where Dx% is the dose delivered to the x% of the BM volume.

### 2.4. Blood Sampling

A full blood count and biochemical analysis one day before the first day of therapy (RT and chemotherapy started on the same day) and every two weeks thereafter was available. The lymphocyte count value recorded after the end of RT was considered for the analysis. This was recorded immediately (for the long RT schedules) or 1 week after the last RT fraction (for short schedules).

### 2.5. Statistical Analysis

A statistical analysis was performed using the PRISM 8 (Graph-Pad Software Inc., San Diego, CA, USA) and the IBM SPSS Statistics 26 (Chicago, IL, USA) software programs. The non-parametric Mann–Whitney test (for two variables) and the Kruskal–Wallis non-parametric test with subsequent Dunn test for intergroup comparison (for multiple variables) was used to compare categorical continuous tumor variables. A linear regression analysis with multicollinearity diagnostics was performed to assess associations between continuous variables. A mixed-model analysis for longitudinal data was also conducted. A *p*-value < 0.05 was used for significance.

## 3. Results

### 3.1. RT Scheme vs. Lymphopenia

**Figure 1:**
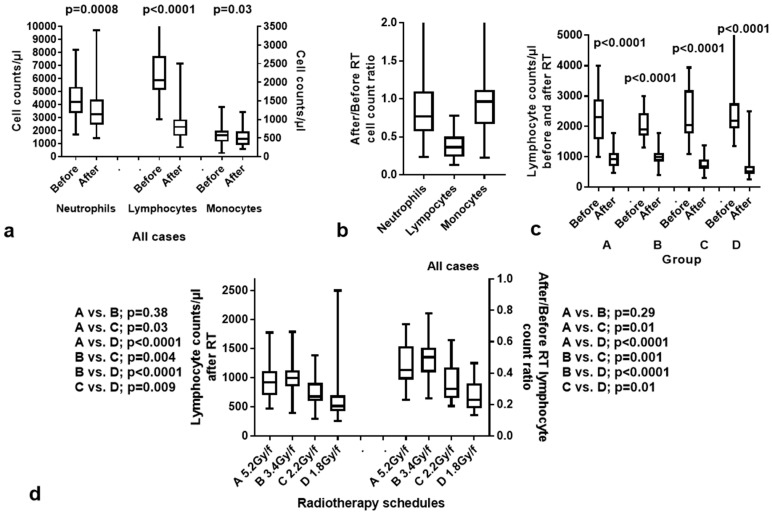
Hematological toxicity after radiotherapy (RT): (**a**) neutrophil, lymphocyte, and monocyte counts before and after RT in all patients; (**b**) graphical representation of the after/before RT ratios of neutrophil, lymphocyte, and monocytes in all patients; (**c**) lymphocyte counts before and after RT according to the group of patients treated with the different RT schedules; (**d**) lymphocyte counts after RT and after/before RT lymphocyte ratios in the four groups of patients. Box and whisker plots show the median value, 25th–75th percentile values, and range.

Overall, RT induced a significant drop in neutrophils (*p* = 0.0008), lymphocytes (*p* < 0.0001), and monocytes (*p* = 0.03); Figure 1a,b. The most striking effect was the induction of lymphopenia (drop in median LCs after RT from 2060 ± 787 to 800 ± 371 counts per μL). Looking into the available data from patients with metastatic colorectal cancer treated with FOLFOX and XELOX chemotherapy (without RT) in our department, we noted no significant lymphotoxicity, suggesting that RT was the main cause of lymphopenia. Moreover, chemotherapy that followed the preoperative CRT schemes in the context of total neoadjuvant therapy did not appear to hinder a gradual recovery of lymphopenia over the subsequent two months.

An analysis of lymphopenia in the groups of patients showed that the 2.2 Gy and 1.8 Gy fractionations (groups C and D) were significantly more lymphotoxic than the 5.2 Gy and 3.4 Gy ones (groups A and B). Figure 1c shows the lymphocyte counts (LCs) before and after RT in the four groups. In all groups, a significant drop in LCs was noted (*p* < 0.0001). There was no significant difference between the LCs before RT between groups (*p* > 0.25). Figure 1d shows the comparison of LCs with *p*-values after RT and of lymphocyte count ratios ‘after/before RT’ in the four groups of patients. The median LCs per μL after RT was 920 ± 342, 1000 ± 293, 675 ± 272, 517 ± 383 in groups A, B, C and D, respectively. Groups A and B had significantly higher LCs after RT than groups C and D. Moreover, conventionally fractionated, yet accelerated, RT (group C) was significantly less lymphotoxic than the standard 1.8 Gy fractionation regimen (group D).

The reduction in neutrophil counts should be considered a result of concurrent chemotherapy and, rather, a numerical finding without any biological or clinical significance. The median granulocyte counts after RT decreased from 4192 ± 1498 to 3250 ± 1586 counts per μL. The lowest 5% percentile value recorded was 1815/μL, which is close to the limits of grade 1 neutropenia (<2000/μL). The drop in monocyte counts, although statistically significant, showed a marginal radiotoxic effect as the median value dropped from 575 ± 228/μL to 470 ± 209/μL.

The overall recovery of LCs was slow. In group A, the median value of LCs increased from 920/μL to 1120/μL and 1300/μL, one and two months after CRT. In group B, the median value increased from 1000/μL to 1100/μL and 1300/μL, one and two months after the end of CRT. The median LCs increased from 675/μL to 1100/μL one month after CRT and remained at 1100/μL at the 2-month time point in patients treated in group C. Thus, 2 months after the end of CRT, LCs remained at the 53%, 68%, and 56% of the baseline levels, in the 2.2 Gy/f, 3.4 Gy/f, and 5.2 Gy/f RT schedules, respectively. Lymphocyte recovery data were not available for group D as the patients had been referred for surgery.

### 3.2. NTD_T vs. Lymphopenia

**Figure 2:**
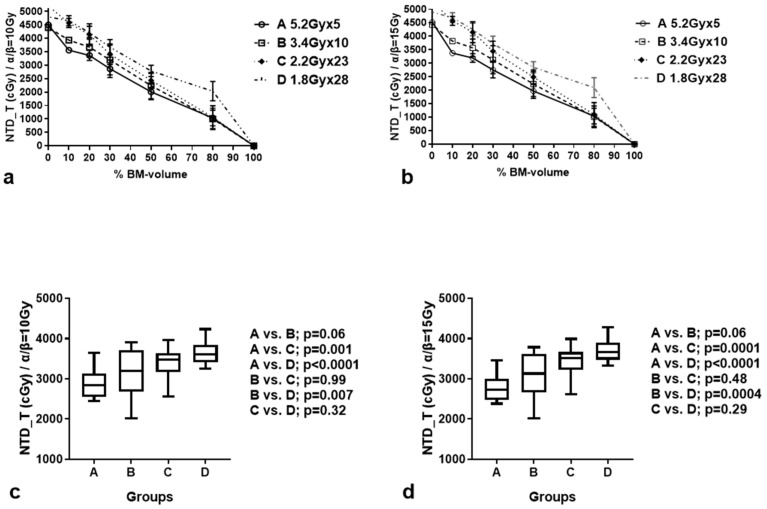
Biological dose (NTD_T)/bone marrow (BM) volume plots in the four groups of patients, for α/β = 10 Gy (**a**) and 15 Gy (**b**). NTD_T received by 30% of the BM volume, calculated for α/β = 10 Gy (**c**) and 15 Gy (**d**), in the four groups of patients. Box and whisker plots show the median value, 25th–75th percentile values, and range.

Table 1 reports the time-corrected NTD_T (±standard deviation) calculated at 10%, 20%, 30%, 50%, and 80% of the BM volume, according to the RT scheme. Figure 2a,b shows the dose/volume plots of the BM for each RT scheme. The NTD_T to the chosen BM volumes gradually increased from groups A to D, whether calculated for α/β = 10 Gy or 15 Gy. Table 2 shows the multiple comparisons. At D30%, the hypofractionated schemes of groups A and B delivered significantly lower NTD_Ts to the BM compared to group D (*p* < 0.01; Figure 2c,d). Moreover, the difference between groups A and C was also significant (*p* < 0.001). There was no significant difference between the NTD/NTD_Ts delivered by the A and B RT schemes (marginally lower D30 in group A; *p* = 0.06), or the B and C RT schemes (*p* > 0.48).

### 3.3. Bone Marrow Exposure vs. Lymphopenia

**Figure 3:**
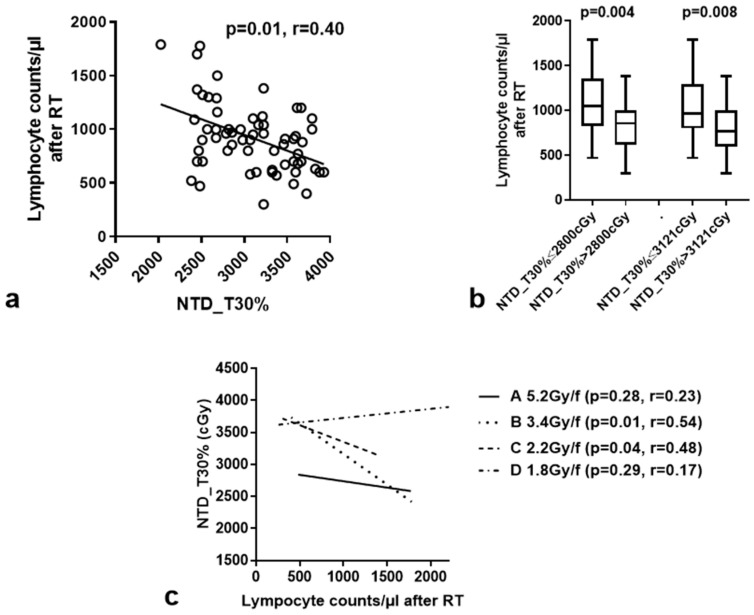
Linear regression analysis of NTD_T (α/β = 15 Gy) delivered to 30% of the bone-marrow volume (NTD_T30%) and lymphocyte counts after RT for all patients (**a**) and for each RT schedule separately (**c**). (**b**) shows the box and whisker plots (median value, 25th–75th percentile values and range) of lymphocyte counts after RT according to two distinct cut-off point of NTD_T30% [33rd percentile (2800 cGy) and the median value (3121 cGy) of NTD_T (all patients) delivered to 30% of the BM].

The normalized total dose without (NTD) and with time correction (NTD_T) (calculated for α/β = 15 Gy and λ = 0.2 Gy/day) for all patients, delivered to different volume percentages of the BM, their correlation with LCs after CRT, and the ‘after/before’ RT lymphocyte count ratio were calculated with a linear regression analysis, and the results are shown in Table 3. A significant inverse association of NTD and NTD_T with LCs after the end of RT was noted for both NTD and NTD_T delivered to 50%, 30%, and 10% of the BM. The best correlation was recorded for the NTD_T 30% (*p* = 0.0003, r = 0.44; Figure 3a). No association with the after/before RT lymphocyte count ratio was noted. In order to test the potential collinearity of NTD_T30% and LC before RT as far as the post-RT LCs are concerned, a bi-variate linear regression analysis with multicollinearity diagnostics was conducted. NDT_T30% and LCs before RT were shown to be independent predictors of post-RT LC (*p* < 0.0001, Tolerance = 0.994, Variance Inflation Factor = 1.006).

We further assessed the correlation of NTD_T30% with the LCs 1 and 2 months after RT (lymphocyte recovery over time). Considering the lymphocytes at the 1- and 2-month time points as the dependent variable, time as the main factor, and NTD_T30%, LCs before RT and post-RT LCs as covariates, it was displayed that LCs before and after RT were significantly correlated with lymphocyte recovery (*p* = 0.001 and *p* < 0.0001, respectively). NTD_T30% was not associated with LC recovery (*p* = 0.49).

Using the 33rd percentile (2800 cGy) and the median value (3121 cGy) of NTD_T (all patients) delivered to 30% of the BM (NTD_T30%) as cut-off points, we noted a significant association of higher NTD_T30% with more intense lymphopenia. Specifically, the median LC was 858/μL vs. 1145/μL when the 33rd percentile of NTD_T30% was applied as a cut-off point (*p* = 0.004), and 770/μL vs. 970/μL, when we used the median value of NTD_T30% as a cut-off point (*p* = 0.008, Figure 3b).

We further assessed the correlation of NTD_T30% with LCs after RT separately in the four fractionation schemes. A significant inverse association was found for the 2.2 Gy/f and 3.4 Gy/f RT schemes (*p* = 0.04, r = 0.48 and *p* = 0.01, r = 0.54, respectively). There was no significant correlation for the 5.2 Gy and 1,8 Gy RT schedules (Figure 3c).

### 3.4. Feasibility of BM Inclusion as an OAR in RT Planning

We further examined the feasibility of RT planning that takes into account the BM as an OAR. Since we found that 30% of the BM volume should receive an NTD_T of less than 28–30 Gy to achieve a better lymphotoxicity profile, we randomly chose nine patients, three from each of the three groups (total of nine patients) treated with VMAT (groups A, B, and C), and we produced a second plan (BM-corrected-1) by inserting the BM structure as an organ at risk (OAR). A third plan (BM-corrected-2) was also performed, after the correction of the target volume to exclude from the PTV areas of BM of the ilium and ischium bones that had been encompassed in the original plan (BM was also considered as an OAR). No corrections were performed for the sacral bone included in the PTV.

The serial cost function was applied to the BM OAR, and the required parameters for this cost function were set as follows: the Equivalent Uniform Dose was set within the range 50%–65% (depends on the plan) of the prescribed physical dose, power law exponent equal to 12, and shrink margin equal to 0.3 cm. Using this constraint, we anticipated that the NTD_T30% to the BM would be less than 30 Gy. The main goal of these plans was for the coverage of PTV to be as close as possible to the original plan, and, furthermore, the three OARs (bladder, sigmoid, and small bowel) should receive a mean and D50% close to the ones noted in the original plans. A percentage dose of less than 50% of their 50% volume was allowed as a maximum limit. If unacceptable plans were to be produced, the power law exponent would be increased to 16 in order to achieve better sparing of BM with a simultaneous acceptable increase in the other two OARs. This latter approach was necessary for patients treated with conventional fractionation, but not for those who received hypofractionated RT. If the plans remained unacceptable, the percentage of physical dose to 50% of the BM volume was planned to gradually increase until the creation of an acceptable plan. However, this has not been necessary in any of the patients herein analyzed.

**Figure 4:**
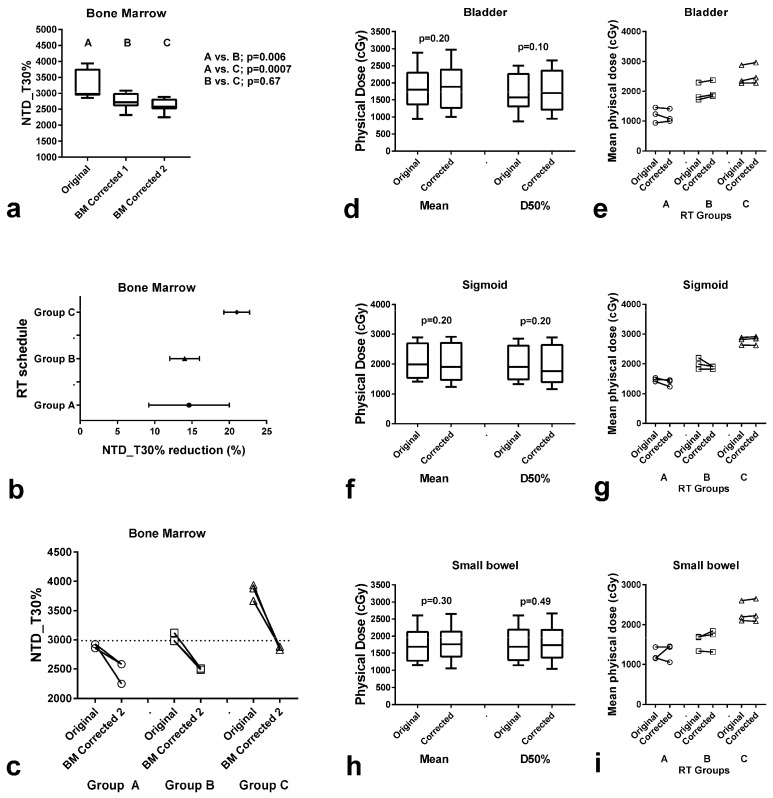
Radiotherapy (RT) dose to the bone marrow (BM) and organs at risk (OARs: bladder, sigmoid, small bowel) after correction of RT planning for BM as an OAR. NTD_T delivered to 30% of the BM (NTD_T30%) before and after correction for the BM (BM-corrected-1 and -2 = without and with removal of the BM of the iliac and ischium bones from the PTV) in all 9 patients (**a**) and according to the RT schedule (**b**,**c**). Box and whisker plots (median, 25th–75th percentile values, and range) in all 9 patients (**d**,**f**,**h**) and individual value plots in each patient group (**e**,**g**,**i**) of the physical dose (mean and at 50% volume–D50%) delivered to the bladder (**d**,**e**), sigmoid (**f**,**g**) and small bowel (**h**,**i**) before and after BM-correction-2.

After creating the original and the corrected plans, we exported the raw data of the cumulative DVH of BM of each chosen patient to an Excel worksheet, and we performed the analysis as previously reported to create the bDVHs for α/β ratio equals to 15 Gy and λ = 0.2 Gy/day. The results are shown in Figure 4. A significant reduction in the NTD_T30% to the BM, ranging from 5.9 to 21.8% (median 8.9%) in the BM-corrected-1 plans, and 9.6–38.2 (median 13.4%) in the BM-corrected-2 plans was noted (*p* = 0.006 and 0.0007, respectively; Figure 4a). The range of the reduction in BM exposure is shown in Figure 4b. The NTD_T threshold of 30 Gy was achieved for all nine patients treated in groups A, B, and C (Figure 4c). The physical dose to the bladder, sigmoid, and small bowel was sustained at the same levels as the ones before the correction for BM (Figure 4d–i).

We further performed a similar analysis applying a 3D-conformal RT plan, using a posterior and two lateral fields with wedges. This was compared to the original VMAT planning. There were no significant differences in the NTD-T30% received by the BM (*p* = 0.74).

**Figure 5:**
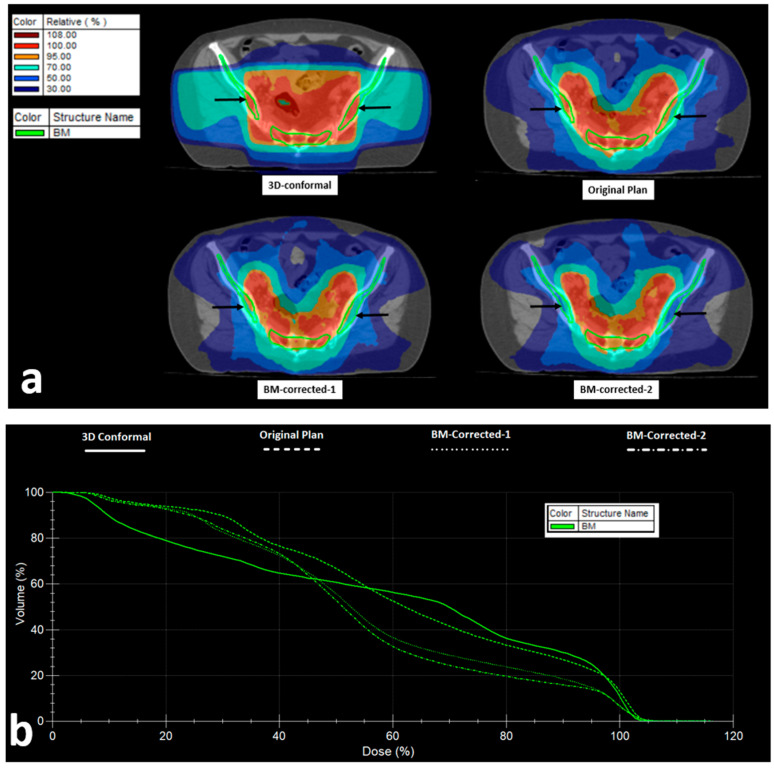
VMAT Radiotherapy planning and bone marrow (BM) dose distribution: (**a**) Isodose areas distribution in 3D-conformal, VMAT (original), and VMAT plans taking into account the bone-marrow as an organ at risk (BM-corrected-1), and VMAT with PTV correction to exclude the BM from iliac and ischium bones (BM-corrected-2) (black arrows show the iliac BM area). (**b**) Dose–volume histograms of the bone marrow in the four abovementioned RT plans.

Figure 5 shows isodose distribution and DVH of the BM of the 3D-conformal, original VMAT, and the BM-corrected-1 and -2 plans.

## 4. Discussion

In recent decades, it has been suggested that effective immune surveillance may drastically prevent or repress tumor growth and can be decisive for the eradication of tumors after chemotherapy and RT [17,18]. This has become an indisputable fact in the modern era of immunotherapy, where complete responses and long disease-free intervals after the blockage of immune checkpoint inhibitory pathways are routine clinical experiences. Immunosuppressive pathways, either active in the tumor microenvironment or at the systemic level, are fundamental for tumor progression. Among the systemic pathways of immune repression, lymphopenia is the most easily detectable. Indeed, pre-treatment lymphopenia, eventually related to patient cachexia or tumor secreted factors, is linked to poor prognosis in colorectal, head–neck cancer, and other malignancies [19].

Unfortunately, treatment-induced lymphopenia is also quite common, further contributing to the pre-existing imbalance between antitumor immunity and cancer. As lymphocytes are among the most radiosensitive cells in the body [20], extended field irradiation that exposes blood, BM, and lymph nodes to high levels of radiation dose frequently promotes lymphopenia, which appears to have a robust influence on the survival outcome of cancer patients, as shown in retrospective studies [7]. Recent data also support the importance of adequate LCs in the outcome of immunotherapy [21]. Cheng et al. reported an analysis of 602 patients with esophageal cancer treated with CRT and anti-PD-1 immunotherapy, showing that low LCs during CRT were an independent factor of poor prognosis [22]. Furthermore, Pasquier et al. displayed that lymphopenia counteracts the efficacy of CRT followed by durvalumab in locally advanced non-small cell lung cancer [23].

The protection of lymphocytes during CRT may therefore be critical for the outcome of RT and, eventually, of immuno-RT. Severe treatment-related lymphopenia in patients with rectal cancer and its eventual impact on treatment outcome have been previously reported in a small number of studies [8,24,25,26]. In this investigation, we retrospectively examined the lymphotoxic effect of different neoadjuvant RT schedules applied for LARC. Long-course CRT and short-course RT are equally acceptable regimens. However, their impact on lymphocytes has not been comparatively examined. An analysis of four RT schedules applying different dose fractionation and overall treatment times (OTT) revealed distinct effects on treatment-induced lymphopenia. Hypofractionated RT (5.2 and 3.4 Gy/f) had a significantly lower lymphotoxic effect. A comparison between two conventionally fractionated regimens also demonstrated that OTT reduction, using a simultaneous integrated boost technique, significantly spared lymphocytes, albeit to a lesser magnitude than hypofractionated and more accelerated regimens.

We hypothesized that the biological radiation dose delivered to the pelvic BM could, in part, explain this big difference between schedules. Kuncman et al. have already shown that the dose–volume parameters concerning active BM as defined in MRI, and total BM as defined in the CT scan predict the degree of lymphopenia in patients treated with CRT for rectal cancer [27]. Moreover, two studies focusing on cervical cancer reported a significant association of the dose with the bone marrow with lymphopenia [28,29]. The radiobiological analysis in this study proved that the NTD_T (assuming an α/β-ratio of 15 Gy and a λ-value of 0.2 Gy/day) delivered to the BM was significantly higher in the most prolonged RT schedule, and decreased gradually in the accelerated conventional and the accelerated hypofractionated schedules.

This finding may explain the differences in terms of lymphotoxicity between the four RT schedules examined. Indeed, a linear regression analysis showed a significant inverse association of NTD_T received by the BM and LCs. The NTD_T delivered to 30% of the BM volume had the strongest correlation. This association was not confirmed in the patients treated with prolonged conventional RT, because most of these patients had eventually received a very high NTD_T (higher than 35 Gy). Similarly, this correlation was not significant in patients treated with the very accelerated 5.2 Gy/f regimen, as the NTD_T delivered to the BM was very low (lower than 30 Gy). Using the median value of the NTD_T (31 Gy) as a cut-off point, we were able to identify two groups of patients with a low vs. high risk of developing lymphopenia.

We therefore suggest that a threshold of NTD_T of 30 Gy delivered to 30% of the BM volume could be used as a constraint during RT planning, eventually leading to a significant sparing of LCs in the blood of patients. Whether the insertion of this constraint in RT planning could be applied without increasing the burden of the radiation dose to OARs like the bladder and small bowel was tested in three cohorts of patients who received accelerated VMAT RT, and had received an NTD_T higher than 30 Gy to 30% of the BM. In all patients, the goal was achieved, following a simple methodology herein described. As presented in the dose/volume histograms, the correction of the PTV to exclude BM areas and subsequent usage of the BM as an organ at risk in treatment planning, provided an evident reduction in the percentage of the radiation dose received by the BM. This achievable reduction falls within the threshold of NTD_T30% demanded to avoid severe lymphopenia, at least for the patients herein retrospectively analyzed. The clinical value of the proposed procedure, however, could be confirmed only in a prospective study, which is already on-going in our department.

The limitations of the current investigation include the retrospective nature of this study and the relatively low number of patients in each fractionation group. In addition, as different chemotherapy schedules have been administered in the four RT groups, an eventual impact on post-RT LCs and recovery cannot be ruled out. Moreover, the recovery of lymphocytes after conventional RT was not available for analysis. Having identified the NTD_T30% dose limit for the bone marrow, we are now running a prospective trial using this constraint in the standard treatment planning of patients, which will eventually provide more robust evidence for the use of this OAR in routine clinical practice, especially in studies focusing on IO/RT combinations.

## 5. Conclusions

This study provides evidence that fractionation and OTT are important parameters defining the lymphotoxic effect of RT in rectal cancer patients. Hypofractionation and treatment acceleration reduce the biological dose delivered to the BM, resulting in significantly higher levels of lymphocytes in the blood of patients after treatment completion. The inclusion of the BM as an OAR and the confinement of NTD_T30% of the BM to values lower than 30 Gy are feasible and should be taken into account to protect the immune system of patients. This could eventually reduce the risk of cancer relapse. This study also brings forward an important suggestion for immuno-RT clinical trials, as hypofractionated and accelerated schemes and carefully designed RT plans sparing the BM may prove critical. The rather unchanged pCR rates obtained by adding immunotherapy to standard neoadjuvant long-course CRT for MMR-proficient cases [5] may be a result of the underestimated role of severe RT-induced lymphopenia. Indeed, early results of the TORCH trial that applied short-course RT with toripalimab displayed surprisingly high pathological complete response rates [30]. We have initiated a prospective trial to assess the proposed RT planning adjustments for their value in preventing lymphopenia in rectal cancer patients undergoing neoadjuvant CRT.

## Figures and Tables

**Table 1:** Time-corrected normalized total dose ± standard deviation (NTD_T ± SD) calculated at 10%, 20%, 30%, 50%, and 80% of the bone marrow (BM) volume, according to the radiotherapy scheme.

Radiotherapy Scheme								
	Group A		Group B		Group C		Group D	
BM Volume (%)	Mean NTD_T (Gy)	±SD	Mean NTD_T (Gy)	±SD	Mean NTD_T (Gy)	±SD	Mean NTD (Gy)	±SD
**α/β = 10 Gy**								
80	10.34	2.90	10.00	3.98	10.63	4.28	20.35	3.61
50	20.09	2.92	22.37	4.88	24.35	2.65	27.84	2.08
30	28.68	3.28	31.92	5.58	34.11	3.82	36.70	2.84
20	33.62	1.90	36.75	3.57	41.36	4.04	42.10	2.39
10	35.58	0.78	39.35	0.93	45.73	1.72	46.56	1.89
**α/β = 15 Gy**								
80	10.44	2.86	10.20	4.00	11.02	4.41	20.99	3.66
50	19.73	2.70	22.29	4.64	24.89	2.64	28.52	2.07
30	27.56	2.95	31.29	5.20	34.50	3.74	37.28	2.78
20	31.97	1.65	35.75	3.29	41.54	3.91	42.57	2.33
10	33.76	0.69	38.22	0.86	45.75	1.66	46.90	1.83

**Table 2:** Multiple comparisons between the NTD_T delivered to different bone marrow volumes (D80%, 50%, 30%, 20%, and 10%), for α/β = 10 Gy and 15 Gy, in the four radiotherapy groups.

Group	D80%	D50%	D30%	D20%	D10%
**α/β = 10 Gy**	***p*-Value**				
A vs. B	>0.9999	0.4597	0.0688	0.2559	0.044
A vs. C	>0.9999	0.0075	0.0018	<0.0001	<0.0001
A vs. D	<0.0001	<0.0001	<0.0001	<0.0001	<0.0001
B vs. C	>0.9999	0.8505	>0.9999	0.0013	0.0006
B vs. D	<0.0001	<0.0001	0.0073	<0.0001	<0.0001
C vs. D	<0.0001	0.0113	0.3275	>0.9999	>0.9999
**α/β = 15 Gy**	***p*-Value**				
A vs. B	>0.9999	0.5234	0.0693	0.2868	0.0366
A vs. C	>0.9999	0.0026	0.0001	<0.0001	<0.0001
A vs. D	<0.0001	<0.0001	<0.0001	<0.0001	<0.0001
B vs. C	>0.9999	0.4167	0.488	0.0006	0.0014
B vs. D	<0.0001	<0.0001	0.0004	<0.0001	<0.0001
C vs. D	<0.0001	0.0099	0.2957	>0.9999	>0.9999

**Table 3:** Linear regression analysis of NTD and NTD_T (α/β = 15 Gy) received by different bone marrow volumes and lymphocyte count parameters (lymphocyte counts after radiotherapy-RT and after/before RT lymphocyte count ratio) for all patients.

	Lymphocyte Counts		After/Before RT Lymphocyte Count Ratio	
	*p*-Value	r-Value	*p*-Value	r-Value
NTD				
80%	0.06	0.23	0.54	0.07
50%	0.003	0.36	0.8	0.01
30%	0.001	0.4	0.88	0.01
10%	0.02	0.29	0.95	0.001
NTD_T				
80%	0.12	0.2	0.76	0.03
50%	0.001	0.4	0.59	0.06
30%	0.0003	0.44	0.31	0.13
10%	0.009	0.32	0.9	0.01

## Data Availability

Research data are stored in an institutional repository and will be shared upon reasonable request to the corresponding author.
